# Interleukin-2 and SOCS-1 proteins involvement in the pathophysiology of severe ovarian hyperstimulation syndrome-a preliminary proof of concept

**DOI:** 10.1186/s13048-014-0106-2

**Published:** 2014-11-26

**Authors:** Raoul Orvieto, Olga Dratviman-Storobinsky, Daniel Lantsberg, Jigal Haas, Roy Mashiach, Yoram Cohen

**Affiliations:** Department of Obstetrics and Gynecology, Chaim Sheba Medical Center (Tel Hashomer), Ramat Gan, Israel; Sackler Faculty of Medicine, Tel Aviv University, Tel Aviv, Israel

**Keywords:** OHSS, Inflammation, Cytokines, Interleukin-2, SOCS, hCG, Pregnancy

## Abstract

**Background:**

Ovarian hyperstimulation syndrome (OHSS), is characterized by marked ovarian enlargement and acute third space fluid sequestration that almost always develops after hCG administration or in early pregnancy. OHSS is similar to vascular leak syndrome (VLS), which may be attributable to the massive increase in systemic inflammatory cytokines. In the present pilot exploratory case series, we sought to evaluate interleukin (IL)-2 and suppressor of cytokine signaling (SOCS)-1 expressions in the peripheral blood mononuclear cells (PBMCs) of patients suffering from severe ovarian hypertimulation syndrome (OHSS), and to examine whether their expressions differ when compared to PBMCs originated from normal early pregnant women (without OHSS).

**Methods:**

Interleukin-2 and SOCS-1 mRNA expressions were examined in PBMCs of 5 women who were hospitalized due to severe OHSS (OHSS group) and 5 women with early IVF pregnancies and without OHSS (control group).

**Results:**

Interleukin-2 mRNA levels in PBMCs were significantly higher in the OHSS as compared to the control groups. Moreover, while SOCS-1 mRNA levels were non-significantly lower, the ratio between IL-2 and SOCS-1 mRNA levels was significantly higher in the OHSS, as compared to the control group.

**Conclusions:**

The inflammatory response to hCG, leading to dysregulation of Il-2 expression and SOCS activation, might be the culprit of OHSS. Additional large prospective studies are required to elucidate the effect of hCG on patients’ inherited inflammatory cascades, which may help discriminating those at risk to develop severe OHSS from those who are not.

## Background

Ovarian hyperstimulation syndrome (OHSS) is a serious complication of ovulation induction, almost always presents either after human chorionic gonadotropin (hCG) administration in susceptible patients or during early pregnancy. Its cardinal features are marked ovarian enlargement and an increase in capillary permeability, with the consequent acute third-space fluid sequestration and its related morbidity [1,2]. Many factors and mediators have been proposed as the intermediate, released by gonadotropin hyperstimulated ovaries at ovulation, which causes the increase in capillary permeability. However, despite many years of clinical experience, the pathophysiology of OHSS is still poorly understood [3].

Interleukin-2 (IL-2), which is produced and secreted by T-helper cells, serves as a central regulator or mediator of the immune response. When administered to human subjects, IL-2 elicited multiple toxic side effects, including “vascular leak syndrome” (VLS), which resembles OHSS. As a consequent of the similarity between VLS and OHSS, we have suggested that the hyperstimulated human ovaries may contain IL-2 which, in turn, might activate the systemic inflammatory response characteristic of OHSS [4].

In the last decades, several evidences have accumulated, suggesting that cytokines stimulation leads to the induction of suppressor of cytokine signaling (SOCS) proteins, which are part of a negative feedback loop, inhibiting cytokines signal transduction that initially led to their production [5]. These regulatory proteins are rapidly transcribed following intracellular Janus kinase-signal transducer and activator of transcription (JAK-STAT) activation, a cascade that governs biological functions, including reproductive processes and cytokine-induced immunological responses [6]. The SOCS family consists of 8 members that are able to antagonize STAT activation and have an important role in cytokines’ balance that determine the profile of T-helper type 1 (Th1)- and Th2-mediated immune responses [7]. Of interest is the SOCS-1 protein, that was demonstrated to be regulated by IL-2 [8], and was also shown to be capable of inhibiting signaling initiated by IL-2 [9].

Inspired by the hypothesis that SOCS-1 might modulate the negative effect of hCG on IL-2 production in OHSS patients, we designed the present pilot exploratory case series, aimed to evaluate the expression of IL-2 and its suppressor, SOCS-1, in the PBMCs of patients suffering from severe OHSS, and to examine whether their expressions differ when compared to PBMCs originated from normal early pregnant women (without OHSS).

## Methods

### Patients

The study population consisted of patients admitted to our gynecology ward, during a 6 months period, due to severe OHSS following an in-vitro fertilization (IVF) treatment. Severe OHSS was defined according to Golan et al. [10] and Navot et al. [1] criteria, while early and late OHSS were defined according to Lyons et al. [11]: 3–7 days and 12–17 days following HCG ovulation triggering, respectively. For the purpose of the study, in addition to the routine follow-up and treatment, blood was drawn from each patient on day 2 of hospitalization, for PBMCs isolation.

The control group consisted of women who have been treated in our IVF unit and conceived. It is our unit policy to measure serum hCG on 13–14 days after embryo transfer (which is performed 3 or 2 days after oocytes retrieval, respectively). If the hCG result reveals a positive pregnancy test (serum hCG levels ≥10 IU/L) a second hCG measurement is performed 2–3 days later. On the day of their second serum hCG measurement, blood was drawn from each patient for PBMCs isolation. The control patients were chosen arbitrarily, from among patients who conceived during the study period and gave their signed informed consent.

The study required no modification of patients routine follow-up or treatment. Informed consent was obtained from all patients before participation in the study, and the study was approved by the institutional Clinical Research Committee, The Sheba Medical Center.

### Peripheral blood mononuclear cells isolation (PBMCs)

Peripheral blood was collected into EDTA tubes. PBMCs were isolated by gradient centrifugation at 2300 RPM for 30 minutes (brake off) using Lymphoprep (Axis-Shield, Oslo, Norway), according to the instructions from the manufacturer. Cells were washed with phosphate-buffered saline (PBS).

### Total RNA extraction and reverse transcription

Total RNA isolation was performed using the RNeasy Micro Kit (Qiagen, Valencia, CA) according to the manufacturer’s instructions. The RNA quality was assessed using a NanoDrop® ND-1000 Spectrophotometer. RNA was transcribed to generate cDNA using qScript™ cDNA Synthesis Kit (Quanta BioSciences) with optimized blend of random hexamers and oligo (dT) primers according to the manufacturer’s instructions.

### Real-time quantitative PCR

Quantification of the mRNA messages coding for SOCS1, IL-2 (gene of interest) and GAPDH (control housekeeping gene) was performed using StepOnePlus™System (Applied Biosystems). Quantitative PCR was performed in a total reaction volume of 20 μl and runs were performed in triplicate. Each 20 μl reaction mix contained 10 μl of a PerfeCTa SYBR Green FastMix (Quanta BioSciences), 0.5-10 pmol of each forward and reverse primer, 2 μl cDNA (made from 0.5 μg RNA) and nuclease-free water to make up the reaction volume. The primers used for real time PCR were as follows:

- GAPDH_F: 5′-GTA TTG GGC GCC TGG TCA-3′
- GAPDH_R: 5′-AGG GGT CAT TGA TGG CAA CA-3′
- IL-2_F: 5′-TCA AAC CTC TGG AGG AAG TGC T-3′
- IL-2_R: 5′-CAT GAA TGT TGT TTC AGA TCC CTT-3′
- SOCS-1_F: 5′-GCG ACT ACC TGA GCT CCT TCC-3′
- SOCS-1_R: 5′-CCA CAT GGT TCC AGG CAA GTA-3′

**Figure 1:**
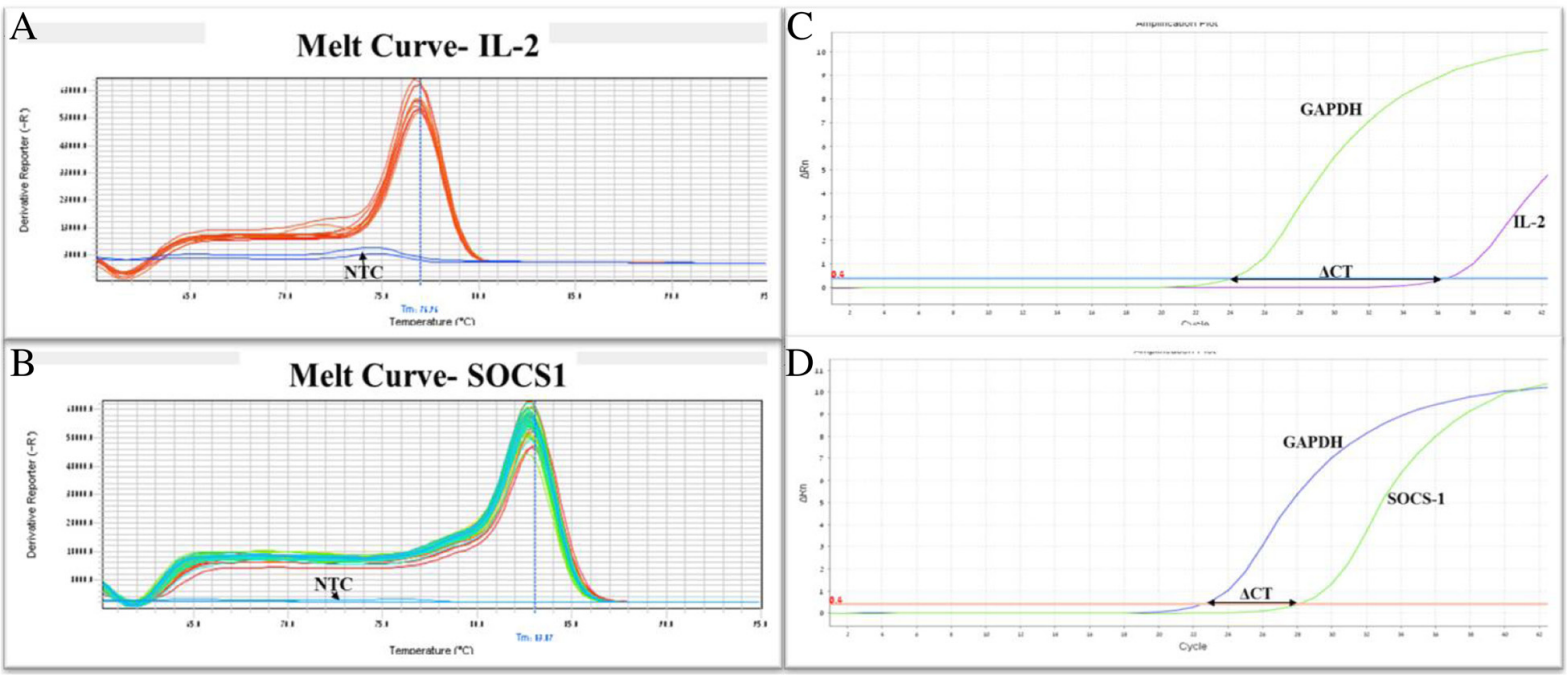
**Amplification time for fluorescence detection and melting curve of PCR product. A**: Melting curve (heat dissociation) analyses for IL-2 gene expression. One PCR product peak at 78°C visible for the target PCR. The negative control shows no visible PCR product. **B**: Melting curve of PCR product for SOCS-1 gene. The real time PCR specificity was determined by the dissociation temperature of PCR products. The single peak at 84°C corresponded to the specific PCR product of SOCS-1 gene. **C–D**: Quantification of IL-2 **(C)** and SOCS-1**(D)** genes, in OHSS patients compared to control. Subsequently the ΔCT for each sample was determined using the equation ΔCT = CTtarget gene – CThousekeeping gene. ΔCT = CT (IL-2) – CT (GAPDH) ΔCT = CT (SOCS-1) – CT (GAPDH).

SYBR green I, a dye which binds to the minor groove of double-stranded DNA (dsDNA) was used as the method of detection. During the various stages of PCR, different intensities of fluorescence signals were detected, depending on the amount of dsDNA present. On completion of the PCR, all PCR products formed were melted to attain a melting curve profile, which enabled the specificity of the reaction to be determined (Figure 1). The melting curve analysis resulted in single product-specific melting temperatures: 87.4°C (GADPH), 89.7°C (SOCS1) and 89.7°C (IL2). No primer primer–dimer formations were generated during the applied 40 real-time PCR amplification cycles. Gene expression levels were calculated by measuring the threshold cycle (CT), an arbitrarily placed threshold which ensures the PCR is in the exponential phase of amplification. The CT is reversely related to the amount of target molecules in the reaction. The comparative CT method is used to calculate the expression level of the gene of interest relative to a reference sample. In this study the analysis was performed using DataAssist™ Software 3.01 (Applied Biosystems).

### Statistical analysis

The results are expressed as means ± standard errors (SE) and medians + ranges. Differences in variables between the two groups were statistically analyzed with Student’s t-test and Wilcoxon rank-sum test, as appropriate. P values of 0.05 or less were considered significant.

## Results

During the study period, 5 women were hospitalized at early gestation and comprised our study group (OHSS group). The control group consisted of 5 women with normal early IVF pregnancies and without OHSS.

The demographic and clinical characteristics of the patients are presented in Table 1. Maternal age, parity, body mass index and cause of infertility were similar among both groups. As expected, the peak estradiol levels on day of hCG administration (9292 ± 2464 pmol/L vs 4167 ± 2730 pmol/L, p < 0.02; respectively) and the number of oocytes retrieved (15.8 ± 2.3 vs 7.8 ± 3.9, p < 0.002; respectively) were significantly higher in the OHSS as compared to the control group.

**Table 1:** **Demographic and clinical characteristics of the study groups**

		**OHSS(5)**	**CONTROL(5)**	**P value**
Age (years)	Mean + SE	32.6 ± 2.3	36.6 ± 5.8	ns
	Median (range)	33 (30–36)	38 (27–41)	
Parity	Mean + SE	0.8 ± 1.3	0.6 ± 0.5	ns
	Median (range)	0 (1–3)	1 (0–1)	
BMI (Kg/m^2^)	Mean + SE	24.2 ± 5.7	24.5 ± 5.6	ns
	Median (range)	24.9 (16.6-32.4)	22.5 (19.7-34.7)	
*Cause of infertility:*				ns
PCO (%)		1(20)	1 (20)	
Mechanical (%)		1(20)	0 (0)	
Male factor (%)		2(40)	3 (60)	
Unexplained (%)		1(20)	1 (20)	
Serum estradiol level on day of hCG administration (pmol/L)	Mean + SE	9292 ± 2464	4167 ± 2730	<0.02
	Median (range)	9336 (6589–11908)	3645 (1153–8575)	
Number of oocytes retrieved	Mean + SE	15.8 ± 2.3	7.8 ± 3.9	<0.002
	Median (range)	16 (12–18)	9 (2–12)	
IL-2 mRNA levels	Mean + SE	2.449 ± 0.255	1.043 ± 0.146	<0.01
	Median (range)	2.272 (1.915-3.403)	1.0 (0.6-1.59)	
SOCS-1 mRNA levels	Mean + SE	0.541 ± 0.144	1.171 ± 0.423	0.22
	Median (range)	0.425 (0.225-0.974)	1.0 (0.285-2.821)	

All the 5 women in our study group conceived via IVF treatments and were hospitalized with late OHSS, occurring 14 ± 2.4 days following hCG administration. The mean hospitalization period during the OHSS episode was 7.2 ± 3.4 days. All the five OHSS patients presented with ascites and with mean hematocrit levels of 43.8 ± 3.0%. One patient had pleural effusion and two others suffered from oliguria. During hospitalization, all patients underwent careful and frequent evaluation and strict monitoring of fluid intake and urine output. IV fluid (crystalloids and colloids) were administered when appropriate. Three patients underwent ultrasound-guided paracentesis of ascitic fluid.

**Figure 2:**
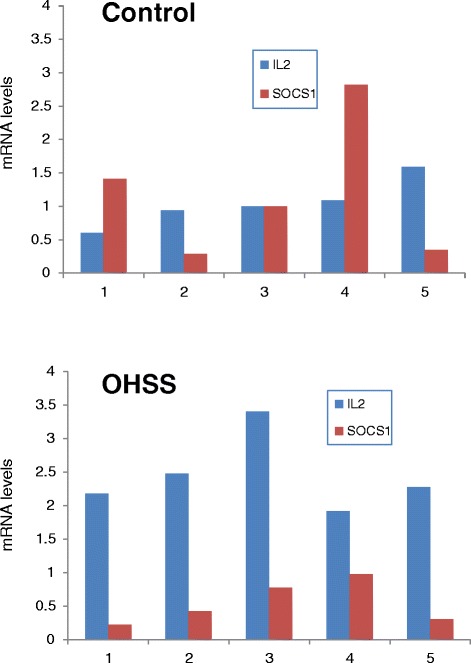
**PBMCs IL-2 and SOCS-1 mRNA levels in the OHSS and control groups.**

Interleukin-2 and SOCS-1 mRNA levels are presented in Table 1 and Figure 2. While IL-2 mRNA levels in PBMCs were significantly higher in the OHSS as compared to the control groups (2.449 ± 0.255 vs 1.043 ± 0.146; p < 0.01, respectively), SOCS-1 mRNA levels were non-significantly lower (0.541 ± 0.144 vs 1.171 ± 0.423; p = 0.22, respectively). Moreover, the ratio between IL-2 and SOCS-1 mRNA levels, was significantly higher in the OHSS as compared to the control group (5.856 vs 1.943; p < 0.04, respectively).

## Discussion

In the present preliminary study, we have demonstrated for the first time, a significantly higher IL-2 mRNA expression in PBMCs of OHSS patients as compared to normal controls. Moreover, the IL-2 to SOCS-1 mRNA expression ratio was also significantly increased, indicating a trend toward T cell activation [12] as a possible culprit to VLS/OHSS.

Together with the accumulating evidence concerning the interaction between the immune and reproductive systems that results from sharing certain lymphohematopoietic cytokines and their receptors [13], several cytokines have been proposed as the intermediate, released by gonadotropin-hyperstimulated ovaries at ovulation, which causes the increased in capillary permeability [3]. However, despite many years of clinical experience, no significant evidence exists to prove neither absolute nor ultimate role of these inflammatory mediators in the pathophysiology of OHSS [3].

Since, OHSS almost always develops after hCG administration or in early pregnancy, it was suggested that the hyperstimulated human ovaries produce and secrete factor(s) that cause OHSS, probably by activating a systemic inflammatory response [3,14]. This notion was based on the similarity of VLS to the clinical picture of OHSS [3,15], the significantly higher cytokine concentration in the follicular fluid at the time of oocyte retrieval in patients in whom OHSS subsequently developed [4,14,16], and the neutrophil and endothelial activations following hCG administration [17–19].

This hypothesis was further substantiated by the observed dose–response modulation of IL-2 production by hCG in short-term primary culture of granulosa cell derived from patients undergoing controlled ovarian hyperstimulation (COH) [20]. While, on the other hand, in vitro studies have demonstrated a negative effect of hCG on PBMCs IL-2 production in normal healthy volunteers [21], or women undergoing COH for IVF without OHSS [22]. This later observations are in line with the growing evidence that hCG may act directly as an immune modulator [23] through its widely distributed receptors in nongonadal tissue, including immune cells, and the observed improvement of symptoms in patients with a Th1-dependent autoimmune disease, during pregnancy [24]. Moreover, animal studies confirmed the immunomodulatory effect of hCG, by downregulating effector cells, including Th1 cells, and suppressing interferon-gamma production [25].

While the aforementioned finding provide support for a modulatory role of hCG in the immune response during pregnancy and strengthens the concept of a distinct regulation of lymphocyte biological activity by hCG, according to our present preliminary findings, the presence of hCG apparently might play a causative role in OHSS, probably by inhibiting SOCS-1 mRNA expression and leading to IL-2 dominance with the consequent activation of the inflammatory response.

Alternatively, despite the small sample size and the inherent disadvantages of this pilot exploratory case series, we observed a different IL-2 to SOCS-1 mRNA expression ratio in PBMCs of patients suffering from severe OHSS, as compared to normal pregnant women without OHSS. This different or aberrant inflammatory response to hCG, observed in OHSS patients, might reflect a tendency toward immune system activation, rather than suppression by hCG, in this particular group of patients.

## Conclusion

Taken together, these data suggest that the normal negative effect of hCG on PBMCs IL-2 production is lost in women that developed OHSS and the resulting dysregulation of IL2 expression and SOCS activation, might be the culprit of OHSS.

We may therefore hypothesize that patients at risk to develop OHSS possess an inherited paradoxical immune response to hCG, with IL-2 instead of SOCS-1 dominance, leading to systemic inflammatory response with the consequent development of OHSS/VLS. Additional large prospective studies are required to elucidate the effect of hCG on patients’ inherited inflammatory cascades, which may help discriminating those at risk to develop severe OHSS from those who are not.

## Abbreviations

COH: Controlled ovarian hyperstimulation
hCG: Human chorionic gonadotropin
IL-2: Interleukin-2
IVF: In vitro fertilization
JAK-STAT: Janus kinase-signal transducer and activator of transcription
OHSS: Ovarian hyperstimulation syndrome
PBMCs: Peripheral blood mononuclear cells
PBS: Phosphate-buffered saline
SOCS: Suppressor of cytokine signaling
Th: T-helper
VSL: Vascular leak syndrome
